# Supplementary material for: Multiomics and cellular senescence profiling of aging human skeletal muscle uncovers Maraviroc as a senotherapeutic approach for sarcopenia
Source: Nat Commun. 2025 Jul 5;16:6207. doi: 10.1038/s41467-025-61403-y (PMC12228793; doi:10.1038/s41467-025-61403-y)
Supplement: Supplementary file 1 — Supplementary Information [file 41467_2025_61403_MOESM1_ESM.pdf]

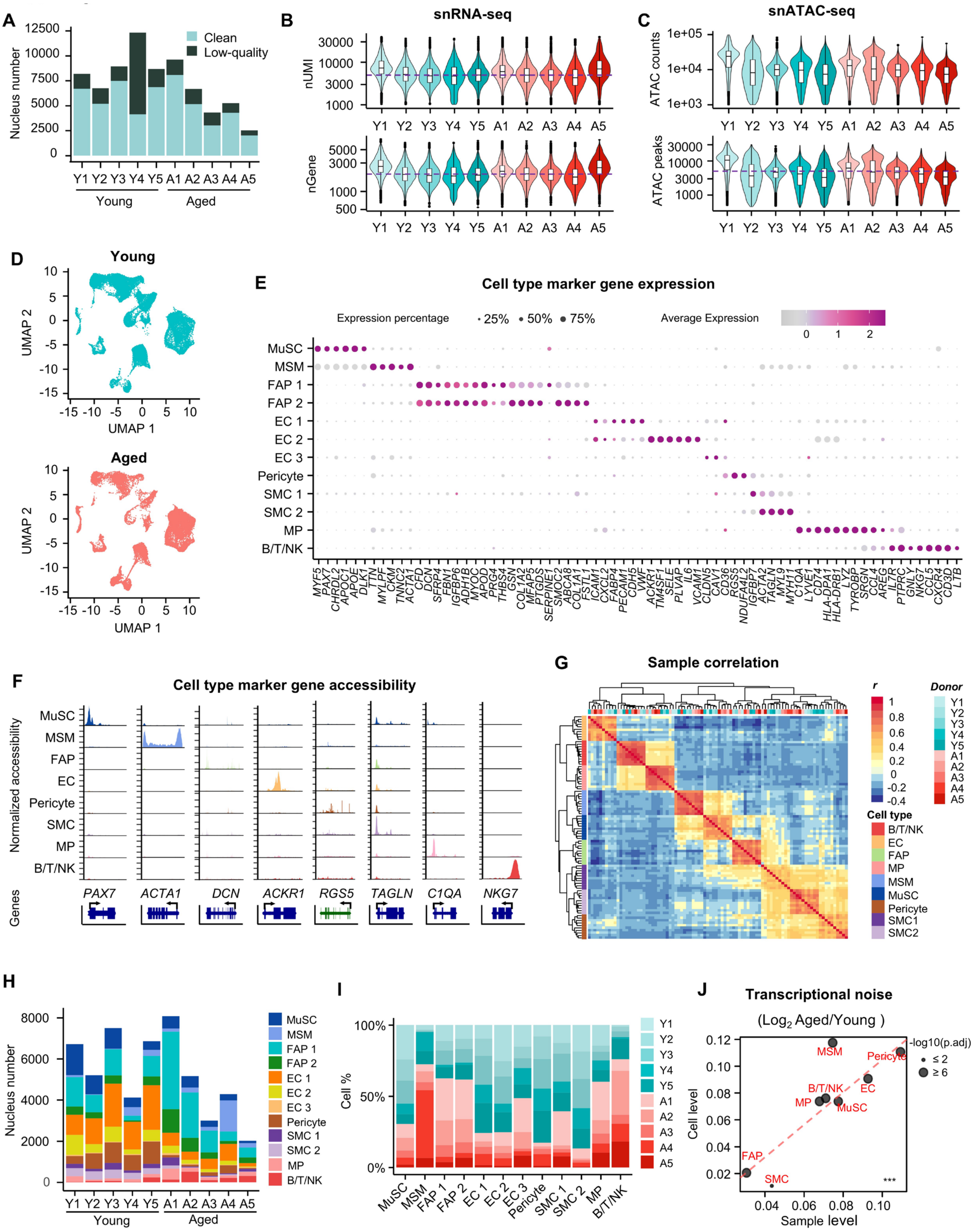
A
Nucleus number
Clean
Low-quality
Y1 Y2 Y3 Y4 Y5 A1 A2 A3 A4 A5
Young
Aged
B
snRNA-seq
nUMI
nGene
C
snATAC-seq
ATAC counts
ATAC peaks
D
Young
Aged
UMAP 1
UMAP 2
E
Cell type marker gene expression
Expression percentage
25%
50%
75%
Average Expression
MuSC
MSM
FAP 1
FAP 2
EC 1
EC 2
EC 3
Pericyte
SMC 1
SMC 2
MP
B/T/NK
MYF5 PAX7 CHRDL2 APOC1 APOE DLK1 TTN MYLPF CKM TNNC2 ACTA1 CFD DCN SFRP4 FBN1 IGFBP6 ADH1B MYOC APOD PRG4 THBS4 GSN COL1A2 MFAP5 PTGDS SERPINE1 SMOC2 ABCA8 COL1A1 FSTL1 ICAM1 CXCL2 FABP4 PECAM1 CDH5 VWF ACKR1 TM4SF1 SELE PLVAP IL6 VCAM1 CLDN5 CAV1 CD36 RGS5 NDUFA4L2 IGFBP7 ACTA2 TAGLN MYL9 MYH11 C1QA LYVE1 CD74 HLA-DPA1 HLA-DRB1 LYZ TYROBP SRGN CCL4 AREG IL7R PTPRC GNLY NKG7 CCL5 CXCR4 CD3D LTB
F
Cell type marker gene accessibility
Normalized accessibility
MuSC
MSM
FAP
EC
Pericyte
SMC
MP
B/T/NK
Genes
PAX7 ACTA1 DCN ACKR1 RGS5 TAGLN C1QA NKG7
G
Sample correlation
r
Donor
Cell type
B/T/NK
EC
FAP
MP
MSM
MuSC
Pericyte
SMC1
SMC2
H
Nucleus number
MuSC
MSM
FAP 1
FAP 2
EC 1
EC 2
EC 3
Pericyte
SMC 1
SMC 2
MP
B/T/NK
Young
Aged
I
Cell %
J
Transcriptional noise
(Log2 Aged/Young)
-log10(p.adj)
≤ 2
≥ 6
Cell level
Sample level
MSM
Pericyte
B/T/NK
EC
MP
MuSC
FAP
SMC
***

Suppl. Fig. 1. Multiomics mapping of senescence atlas in aging human muscle. A Bar plot showing nucleus number from each human young (Y) or aged(A) donor skeletal muscle sample. **B** Box plot showing the unique molecular identifiers (UMIs) (top) and the number of genes (bottom) detected in each nucleus by analyzing snRNA-seq data. The dashed lines indicate 5000 UMIs and 2000 genes, respectively. **C** Box plot showing the number of ATAC counts and the number of peaks detected in each nucleus by analyzing the snATAC-seq data. The dashed line indicates the level of 5000 ATAC peaks. **D** UMAP visualization of young and aged groups showing no obvious batch effect. **E** Dot plot showing the snRNA-seq derived normalized expression levels of representative marker genes for each cell (sub)type. **F** snATAC-seq derived chromatin accessibility of representative marker genes (indicated below) for each cell type. **G** Heatmap showing the Pearson correlation of pseudo-bulked cell type-specific expression profiles among samples. **H** Bar plot showing the number of nuclei of each cell (sub)type captured in each of the young or aged samples. **I** Stacked bar plot showing the percentage of each sample in each of the 12 cell (sub)types. Colors used for indicating cell type are consistent with main figures. **J** Scatter plot showing the $\log_2$ ratio of transcriptional noise between aged and young samples as calculated using sample averages (n = 10) and single cells on the X and Y axes, respectively. The dot size correlates with the negative $\log_{10}$ adjusted p-value of the cell type-level differential transcriptional noise analyzed using Wilcoxon test and the red dashed line corresponds to the robust F-test for comparing two linear models. Source data are provided as a Source Data file.

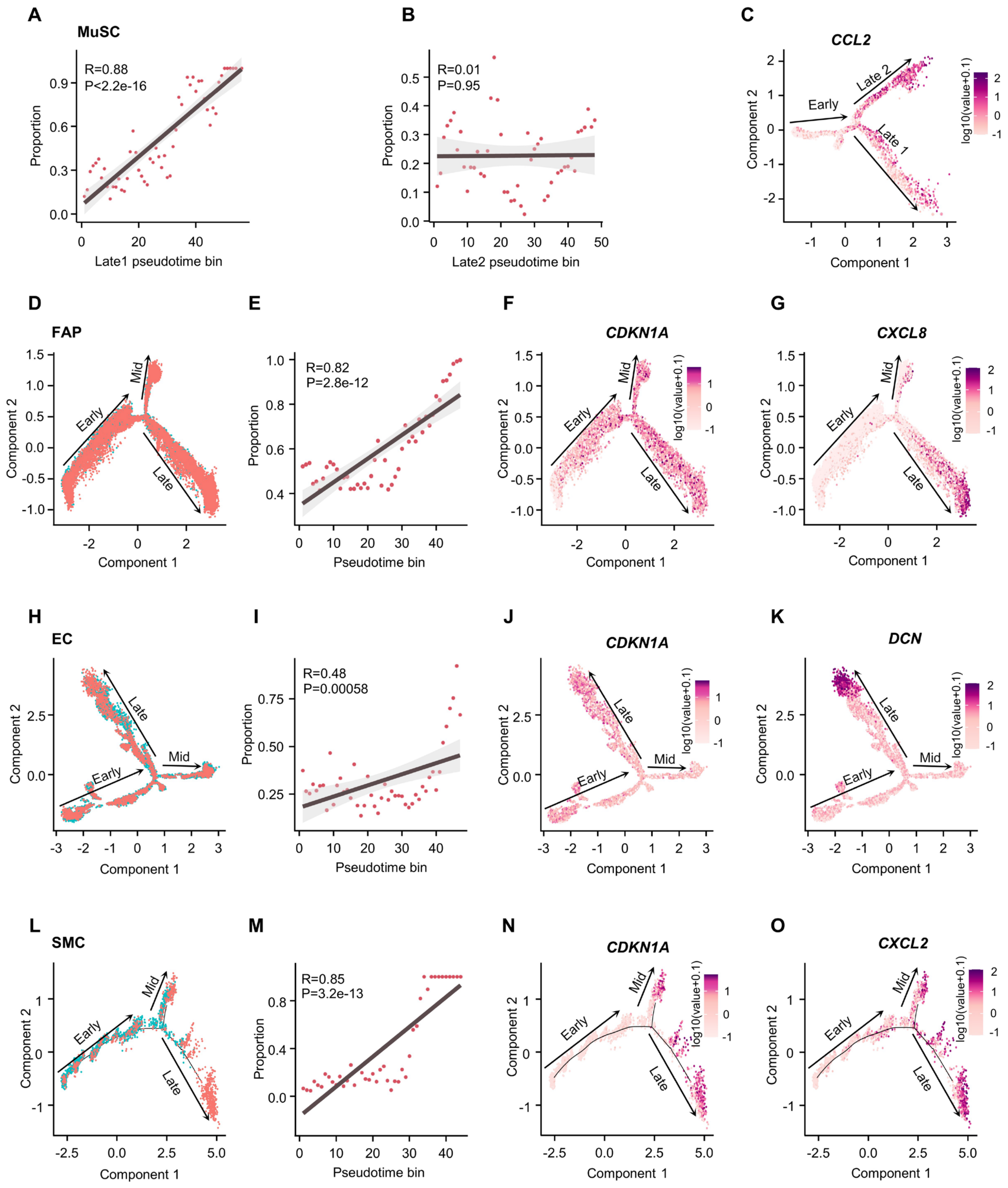
A
MuSC
R=0.88
P<2.2e-16
Proportion
Late1 pseudotime bin
B
R=0.01
P=0.95
Proportion
Late2 pseudotime bin
C
CCL2
Early
Late 2
Late 1
Component 2
Component 1
log10(value+0.1)
D
FAP
Early
Mid
Late
Component 2
Component 1
E
R=0.82
P=2.8e-12
Proportion
Pseudotime bin
F
CDKN1A
G
CXCL8
H
EC
I
R=0.48
P=0.00058
J
CDKN1A
K
DCN
L
SMC
M
R=0.85
P=3.2e-13
N
CDKN1A
O
CXCL2

Suppl. Fig. 2. Heterogeneity and dynamics of cellular senescence in aging human muscle. A Scatter plot showing the proportion of aged MuSCs along the Early-Late1 pseudotime in 52 time bins (sized 0.1 per time bin). Pearson correlation of the proportion of aged nuclei and pseudotime: $R = 0.87$ and $P < 2.2 \times 10^{-16}$ (two-sided), with 95% CI (gray) shown. **B** Scatter plot showing the proportion of aged MuSCs along the Early-Late2 pseudotime in 47 time bins (sized 0.1 per time bin). Pearson correlation: $R = 0.01$ and $P = 0.95$ (two-sided), with 95% CI (gray) shown. **C** Expression level of *CCL2* gene projected into discriminative dimensionality reduction (DDR) tree visualization of MuSCs. **D** DDR tree visualization of FAP trajectory with mapping of age group information. **E** Scatter plot showing the proportion of aged FAPs along the Early-Late pseudotime bins (47 time bins, sized 0.15 per time bin). Pearson correlation: $R = 0.82$ and $P = 2.8 \times 10^{-12}$ (two-sided), with 95% CI (gray) shown. **F-G** Expression levels of *CDKN1A* and *CXCL8* genes projected into DDR tree visualization of FAPs. **H** DDR tree visualization of EC trajectory with mapping of age group information. **I** Scatter plot showing the proportion of aged ECs along the Early-Late pseudotime bins (47 time bins, sized 0.20 per time bin). Pearson correlation: $R = 0.48$ and $P = 5.8 \times 10^{-4}$ (two-sided), with 95% CI (gray) shown. **J-K** Expression levels of *CDKN1A* and *DCN* gene projected into DDR tree visualization of ECs. **L** DDR tree visualization of SMC trajectory with mapping of age group information. **M** Scatter plot showing the proportion of aged SMCs along the Early-Late pseudotime bins (44 time bins, sized 0.20 per time bin). Pearson correlation: $R = 0.85$ and $P = 3.2 \times 10^{-13}$ (two-sided), with 95% CI (gray) shown. **N-O** Expression levels of *CDKN1A* and *CXCL2* genes projected into DDR tree visualization of SMCs. Source data are provided as a Source Data file.

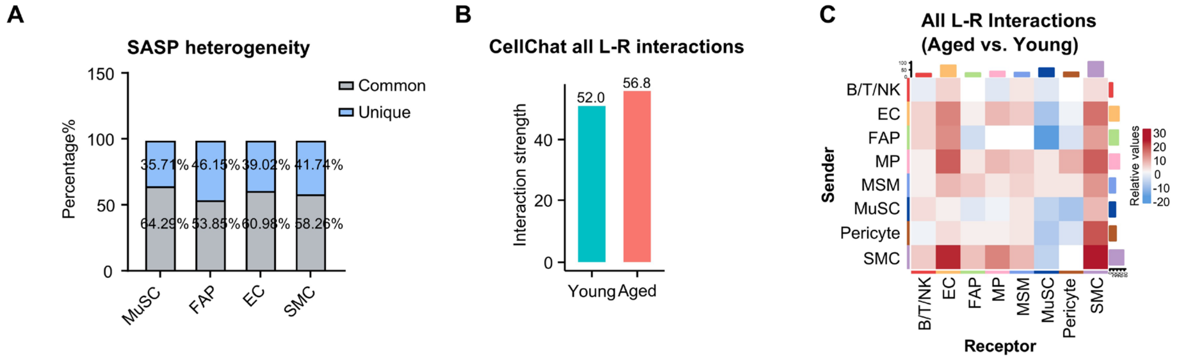

Suppl. Fig. 3. SASP profiling and function in senescent cells. **A** Bar plot showing the ratios of common and cell type specific SASPs in MuSCs, FAPs, ECs and SMCs. **B** Bar plot comparing the interaction strength of all Ligand (L)-Receptor (R) mediated intercellular communications in aged vs. young group. **C** Heatmap showing differential number of all L-R mediated interactions between two cell types. Red/Blue represents increased/decreased signaling in the aged vs. young. The colored bar plot on the top or right represents the incoming/outgoing signaling setting each cell type as receptor/sender (sum of column/row of displayed values). Source data are provided as a Source Data file.

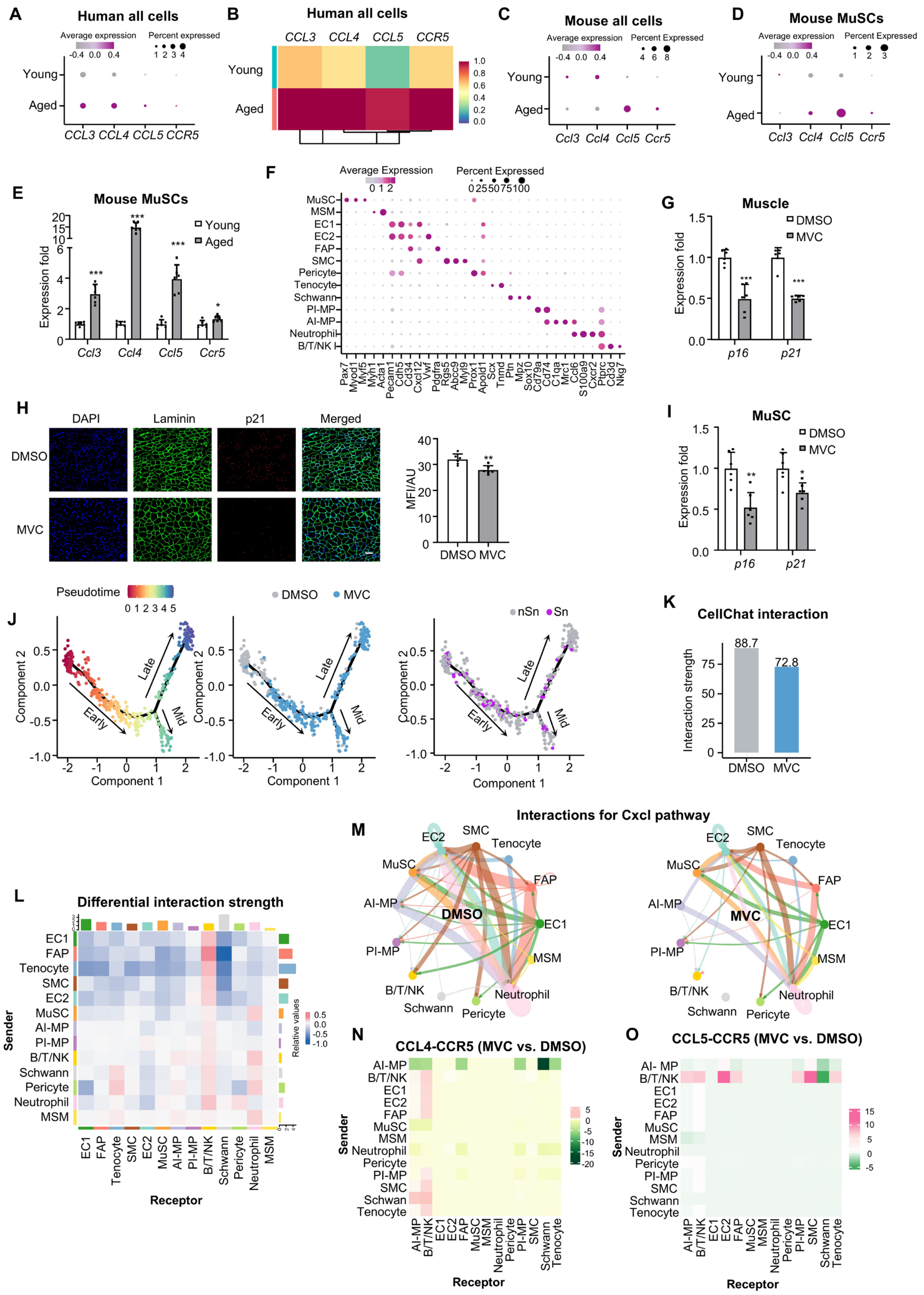
A
Human all cells
Average expression
Percent expressed
Young
Aged
CCL3 CCL4 CCL5 CCR5
B
Human all cells
CCL3 CCL4 CCL5 CCR5
Young
Aged
C
Mouse all cells
Average expression
Percent Expressed
Young
Aged
Ccl3 Ccl4 Ccl5 Ccr5
D
Mouse MuSCs
Average expression
Percent Expressed
Young
Aged
Ccl3 Ccl4 Ccl5 Ccr5
E
Mouse MuSCs
Young
Aged
Expression fold
Ccl3 Ccl4 Ccl5 Ccr5
F
Average Expression
Percent Expressed
MuSC
MSM
EC1
EC2
FAP
SMC
Pericyte
Tenocyte
Schwann
PI-MP
AI-MP
Neutrophil
B/T/NK
Pax7 Myod1 Myf5 Myh1 Acta1 Pecam1 Cdh5 Cd34 Cxcl12 Vwf Pdgfra Rgs5 Abcc9 Myl9 Prox1 Apod1 Scx Tnmd Ptn Mpz Sox10 Cd79a Cd74 C1qa Mrc1 Ccl6 S100a9 Cxcr2 Ptprc Cd3d Nkg7
G
Muscle
DMSO
MVC
Expression fold
p16 p21
H
DAPI
Laminin
p21
Merged
DMSO
MVC
MFI/AU
DMSO MVC
I
MuSC
DMSO
MVC
Expression fold
p16 p21
J
Pseudotime
DMSO
MVC
nSn
Sn
Component 2
Component 1
Early
Late
Mid
K
CellChat interaction
Interaction strength
88.7
72.8
DMSO MVC
L
Differential interaction strength
Sender
Receptor
Relative values
EC1
FAP
Tenocyte
SMC
EC2
MuSC
AI-MP
PI-MP
B/T/NK
Schwann
Pericyte
Neutrophil
MSM
M
Interactions for Cxcl pathway
DMSO
MVC
EC2
SMC
Tenocyte
MuSC
FAP
AI-MP
EC1
PI-MP
MSM
B/T/NK
Neutrophil
Schwann
Pericyte
N
CCL4-CCR5 (MVC vs. DMSO)
Sender
Receptor
AI-MP
B/T/NK
EC1
EC2
FAP
MuSC
MSM
Neutrophil
Pericyte
PI-MP
SMC
Schwan
Tenocyte
O
CCL5-CCR5 (MVC vs. DMSO)
Sender
Receptor
AI- MP
B/T/NK
EC1
EC2
FAP
MuSC
MSM
Neutrophil
Pericyte
PI-MP
SMC
Schwann
Tenocyte

Suppl. Fig. 4. Maraviroc is a potential senotherapeutic for sarcopenia. A Dot plot showing snRNA-seq derived expression of *CCR5* axis genes in all human muscle mononuclear cells. **B** Heatmap showing the pseudo-bulked expression of *CCR5* axis genes in all mononuclear cells of young or aged muscle. **C-D** Dot plot showing snRNA-seq derived expression of *Ccr5* axis genes in published murine muscle mononuclear cells (C) and MuSCs (D). **E** RT-qPCR detection of *Ccr5* axis in young/aged MuSCs, n=6. *p*=0.0024 (*Ccl3*), 0.00048 (*Ccl4*), 0.0084 (*Ccl5*), 0.0047 (*Ccr5*). **F** Dot plot showing the expression signatures of representative marker genes for each cell type. **G** RT-qPCR detection of *p16* and *p21* expression in DMSO/MVC treated muscles, n=6. *p*=0.000088 (*p16*), 0.0000017 (*p21*). **H** IF staining of P21 and Laminin were performed on the above treated muscle sections. The Main Fluorescence Intensity (MFI) of P21 is shown on the right. Scale bar: 50 μm, n=6. *p*=0.0034. **I** RT-qPCR detection of *p16* and *p21* expression in the above treated muscles and MuSCs, n=6. *p*=0.0016 (*p16*), 0.011 (*p21*). **J** DDR tree visualization of MuSC trajectory with mapping of pseudotime (left), treatment group (mid) and senescence annotation (right). **K** Bar plot showing the interaction strength of intercellular communications calculated by CellChat in DMSO or MVC group. **L** Heatmap showing differential number of SASP-mediated interactions between two cell types. Red/Blue represents increased/decreased signaling in the MVC vs. DMSO. The colored bar plot on the top or right represents the incoming/outgoing signaling setting each cell type as receptor/sender (sum of column/row of displayed values). **M** Circle plot showing the signal strength change by aggregating all L-R pairs within Cxcl pathway. The edge colors correspond to the sender cell types, and the edge weights are proportional to the interaction strength. **N-O** Heatmap showing the interaction frequency of Ccl4-Ccr5 and Ccl5-Ccr5 across different cell types in MVC vs. DMSO. All the bar graphs are presented as mean + SD, unpaired two-sided Student's *t*-test was used to calculate the statistical significance (E, G-I): $^{*}p < 0.05$, $^{**}p < 0.01$, $^{***}p < 0.001$, *n.s.* = no significance. Source data are provided as a Source Data file.

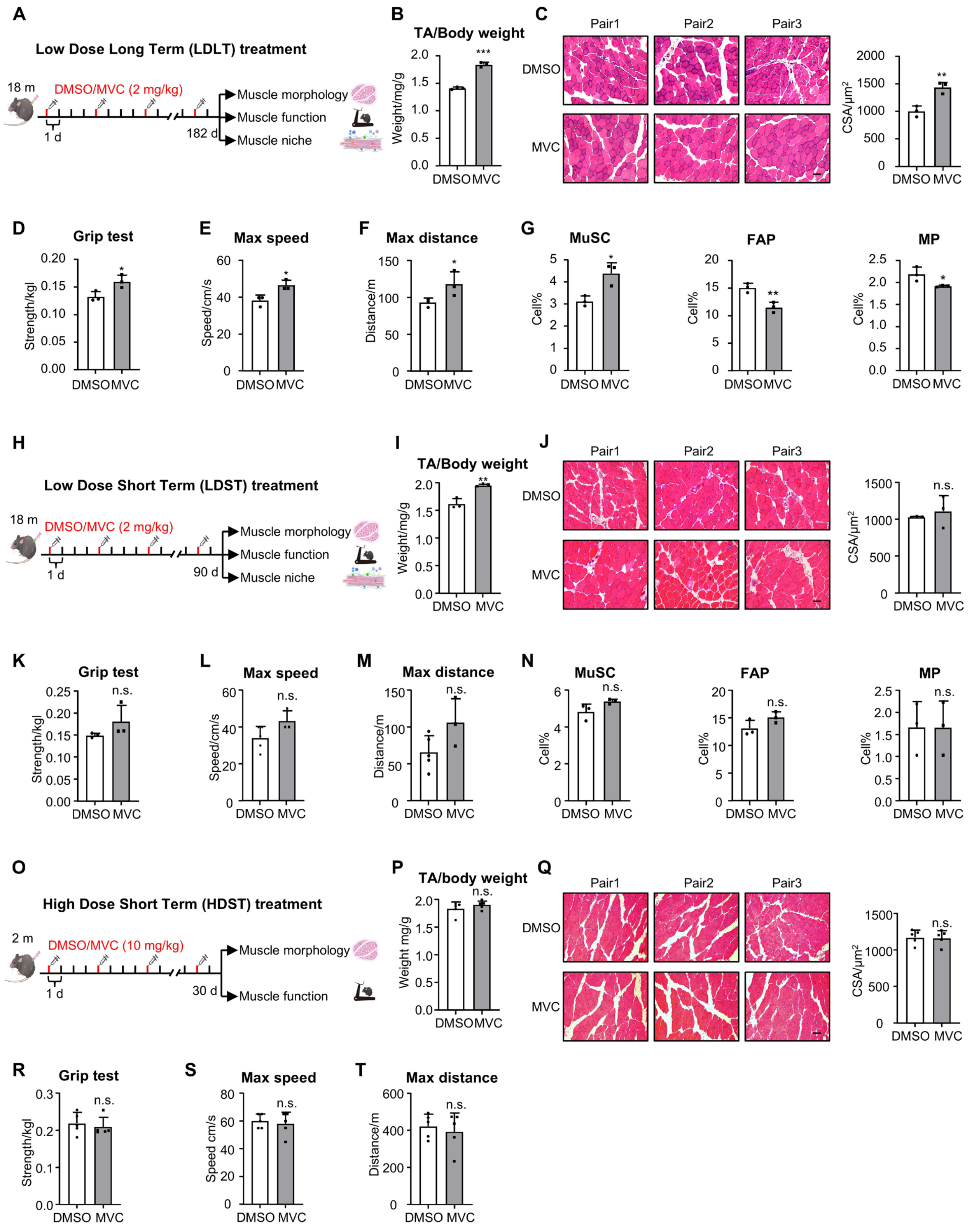
A
Low Dose Long Term (LDLT) treatment
18 m
DMSO/MVC (2 mg/kg)
1 d
182 d
Muscle morphology
Muscle function
Muscle niche
B
TA/Body weight
C
Pair1
Pair2
Pair3
DMSO
MVC
D
Grip test
E
Max speed
F
Max distance
G
MuSC
FAP
MP
H
Low Dose Short Term (LDST) treatment
18 m
DMSO/MVC (2 mg/kg)
1 d
90 d
I
TA/Body weight
J
K
Grip test
L
Max speed
M
Max distance
N
MuSC
FAP
MP
O
High Dose Short Term (HDST) treatment
2 m
DMSO/MVC (10 mg/kg)
1 d
30 d
P
TA/body weight
Q
R
Grip test
S
Max speed
T
Max distance

Suppl. Fig. 5. Additional treatment regime of Maraviroc for sarcopenia. A Schematic of low dose long term treatment/assessment regime of MVC effect in aging muscle. Created in BioRender. Li, Y. (2025) https://BioRender.com/xsxka6w **B** The ratio of TA/body weight of the treated mice, n=3. p=0.000078. **C** H&E staining and quantification of cross-sectional areas (CSAs) of tibialis anterior (TA) muscles collected from the above-treated mice. Scale bar: 50 µm, n=3. p=0.0063. **D** The treated mice were subject to a grip strength meter for strength measurement, n=3. p=0.0032. **E-F** The treated mice were subject to treadmill exercise and the maximal running speed and distance were recorded, n=3. p=0.076 (E), 0.024 (F). **G** Flow cytometry detection of the percentages of MuSC, MP and FAP populations in the treated mice, n=3. p=0.047 (MP), 0.0086 (FAP), 0.017 (MuSC). **H** Schematic of low dose short term treatment/assessment regime of MVC effect in aging muscle. Created in BioRender. Li, Y. (2025) https://BioRender.com/xsxka6w **I** The ratio of TA/body weight of the treated mice, n=3. p=0.0044. **J** H&E staining and quantification of CSAs of TA muscles collected from the above-treated mice. Scale bar: 50 µm, n=3. **K** The treated mice were subject to a grip strength meter for strength measurement, n=3. **L-M** The treated mice were subject to treadmill exercise; the maximal running speed and distance were recorded, n=3. **N** Flow cytometry detection of the percentages of MuSC, MP, and FAP populations in the treated mice, n=3. **O** Schematic of high dose short term (one-month) treatment/assessment regime of MVC effect in 2-month-old young muscle. Created in BioRender. Li, Y. (2025) https://BioRender.com/xsxka6w **P** The ratio of TA/body weight of the treated mice, n=5. **Q** H&E staining and quantification of CSAs of TA muscles collected from the above-treated mice. Scale bar: 50 µm, n=5. **R** The treated mice were subject to a grip strength meter for strength measurement, n=5. **S-T** The treated mice were subject to treadmill exercise and the maximal running speed and distance were recorded, n=5. All the bar graphs are presented as mean + SD, unpaired two-sided Student's *t*-test was used to calculate the statistical significance (B-G, I-N, P-T): *p < 0.05, **p < 0.01, ***p < 0.001, *n.s.* = no significance. Source data are provided as a Source Data file.

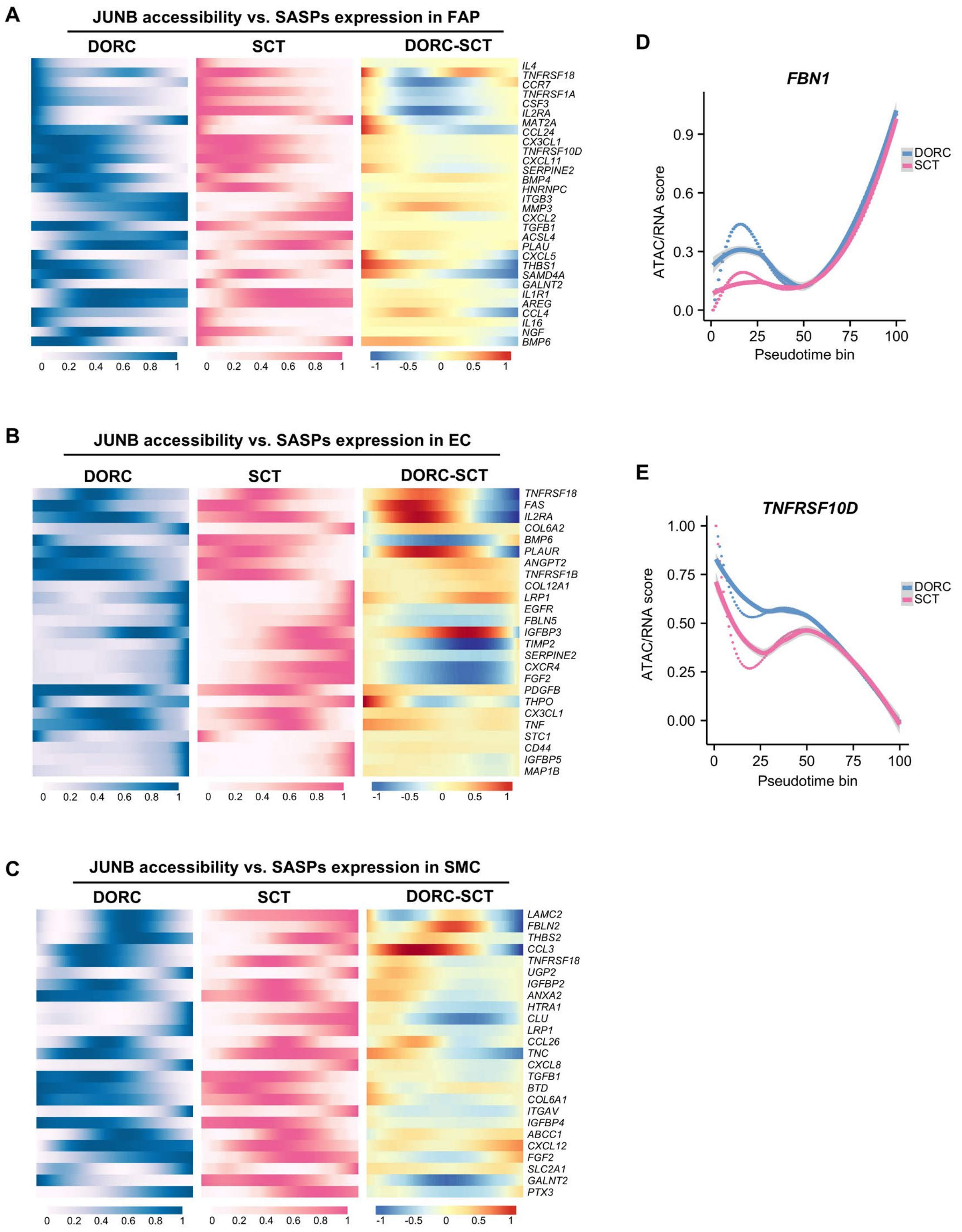
A
JUNB accessibility vs. SASPs expression in FAP
DORC
SCT
DORC-SCT
IL4
TNFRSF18
CCR7
TNFRSF1A
CSF3
IL2RA
MAT2A
CCL24
CX3CL1
TNFRSF10D
CXCL11
SERPINE2
BMP4
HNRNPC
ITGB3
MMP3
CXCL2
TGFB1
ACSL4
PLAU
CXCL5
THBS1
SAMD4A
GALNT2
IL1R1
AREG
CCL4
IL16
NGF
BMP6
0 0.2 0.4 0.6 0.8 1
0 0.2 0.4 0.6 0.8 1
-1 -0.5 0 0.5 1
B
JUNB accessibility vs. SASPs expression in EC
DORC
SCT
DORC-SCT
TNFRSF18
FAS
IL2RA
COL6A2
BMP6
PLAUR
ANGPT2
TNFRSF1B
COL12A1
LRP1
EGFR
FBLN5
IGFBP3
TIMP2
SERPINE2
CXCR4
FGF2
PDGFB
THPO
CX3CL1
TNF
STC1
CD44
IGFBP5
MAP1B
0 0.2 0.4 0.6 0.8 1
0 0.2 0.4 0.6 0.8 1
-1 -0.5 0 0.5 1
C
JUNB accessibility vs. SASPs expression in SMC
DORC
SCT
DORC-SCT
LAMC2
FBLN2
THBS2
CCL3
TNFRSF18
UGP2
IGFBP2
ANXA2
HTRA1
CLU
LRP1
CCL26
TNC
CXCL8
TGFB1
BTD
COL6A1
ITGAV
IGFBP4
ABCC1
CXCL12
FGF2
SLC2A1
GALNT2
PTX3
0 0.2 0.4 0.6 0.8 1
0 0.2 0.4 0.6 0.8 1
-1 -0.5 0 0.5 1
D
FBN1
ATAC/RNA score
0.0
0.3
0.6
0.9
DORC
SCT
0
25
50
75
100
Pseudotime bin
E
TNFRSF10D
ATAC/RNA score
0.00
0.25
0.50
0.75
1.00
DORC
SCT
0
25
50
75
100
Pseudotime bin

Suppl. Fig. 6. Defining TFs governing senescence state and SASP induction in human muscle. A-C Heatmaps highlighting smoothed normalized JUNB DORC accessibility, SCT-normalized RNA expression, and the level of difference (DORC-RNA) for JUNB-target SASP genes identified to be significantly associated with FAP (A), EC (B), and SMC (C) aging pseudotime. **D-E** JUNB Chromatin (DORC) versus normalized gene expression (RNA) dynamics of SASP genes *FBN1* (D) and *TNFRSF10D* (E) with respect to MuSC aging pseudotime. The dotted line represents a LOESS fit to the JUNB DORC accessibility/gene expression dynamics along pseudotime in a gene-wise manner, derived from smooth spline curves fitted to the values obtained from 100 pseudotime bins. Source data are provided as a Source Data file.

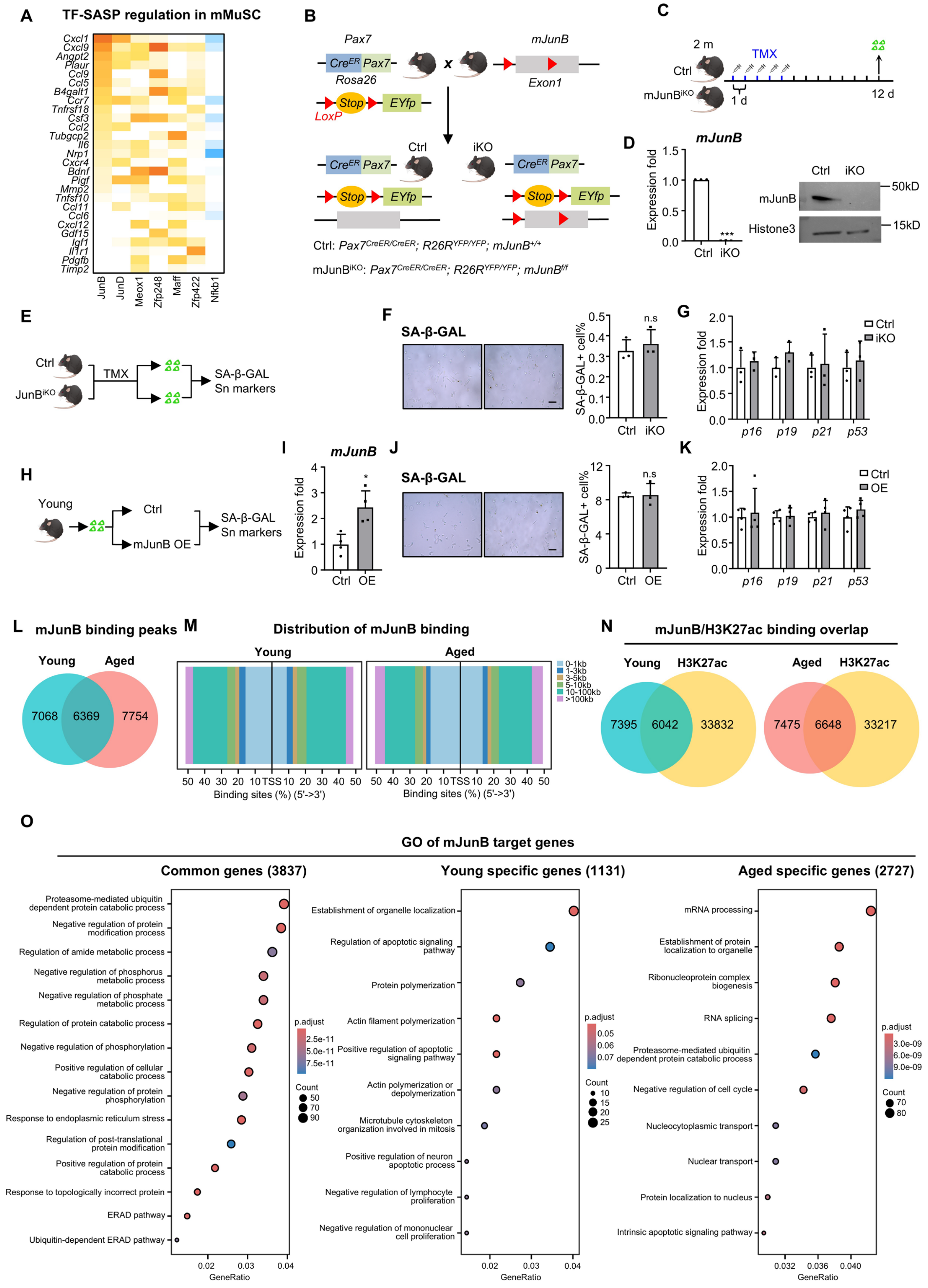

A
TF-SASP regulation in mMuSC
B
C
D
mJunB
E
F
SA-β-GAL
G
H
I
mJunB
J
SA-β-GAL
K
L
mJunB binding peaks
M
Distribution of mJunB binding
N
mJunB/H3K27ac binding overlap
O
GO of mJunB target genes
Common genes (3837)
Young specific genes (1131)
Aged specific genes (2727)

Suppl. Fig. 7. JUNB activates SASP induction in senescent MuSCs via enhancer regulation. A Heatmap showing snATAC-seq detected DORC regulation scores for top-ranked TF-SASP association in mouse MuSCs. **B** Breeding scheme for generating inducible JunB conditional knock out (JunB^{iKO}: *Pax7*$^{CreERT2/R26YFP}$; *JunB*$^{f/f}$) and Control (Ctrl: *Pax7*$^{CreERT2/R26Yfp}$; *JunB*$^{+/+}$) mice. **C** Schematic of the experimental design for inducing JunB specific knocking out in MuSCs by Tamoxifen (TMX) injection. **D** RT-qPCR and western blot detection of the RNA and protein expression levels of JunB in Ctrl vs. iKO MuSCs, n=3. p=0.00000018. **E** Schematic of the experimental design for analyzing senescent MuSCs from Ctrl and JunB-iKO mice. **F** SA-β-GAL staining showing the percentage of senescent MuSCs in Ctrl vs. iKO. Scale bar: 50 μm, n=3. **G** RT-qPCR detection of the expression levels of senescent marker genes in Ctrl and iKO MuSCs, n=3. **H** Schematic of the experimental design for analyzing senescence in MuSCs with JunB overexpression. **I** RT-qPCR confirmation of the *JunB* overexpression after transfecting the JunB overexpressing or Ctrl plasmid into the MuSCs, n=3. p=0.010. **J** SA-β-GAL staining showing the senescent MuSCs in the above cells. Scale bar: 50 μm, n=3. **K** RT-qPCR detection of the expression levels of senescent marker genes in the above cells, n=3. **L** Pie chart showing the overlapping of JunB CUT&RUN-seq identified binding peaks in young and aged MuSCs. **M** Stacked bar chart showing the feature distribution of JunB binding sites based on the distance to TSS sites. **N** Pie chart showing the overlapping of JunB binding with H3K27ac in both young and aged MuSCs. **O** GO analysis of JunB target genes in young and aged MuSCs. The common and young or aged unique genes were shown respectively. All the bar graphs are presented as mean + SD, unpaired two-sided Student's t-test was used to calculate the statistical significance (D, F-G, I-K): $*p < 0.05$, $**p < 0.01$, $***p < 0.001$, *n.s.* = no significance. Source data are provided as a Source Data file.
