# Supplementary material for: Multiomics and cellular senescence profiling of aging human skeletal muscle uncovers Maraviroc as a senotherapeutic approach for sarcopenia
Source: Nat Commun. 2025 Jul 5;16:6207. doi: 10.1038/s41467-025-61403-y (PMC12228793; doi:10.1038/s41467-025-61403-y)
Supplement: Supplementary file 2 — Description of Additional Supplementary Files [file 41467_2025_61403_MOESM2_ESM.pdf]

Description of Additional Supplementary Files

Supplementary Datasets

Supplementary Data 1. Human skeletal muscle donor information

Supplementary Data 2. snRNA-seq analysis of the single-nuclei multiome data in aging human skeletal muscle

Supplementary Data 3. DEGs and GO analysis from snRNA-seq data

Supplementary Data 4. Analysis of SASP atlas and cell-cell communications in aging human skeletal muscle

Supplementary Data 5. Single-cell RNA-seq profiling in DMSO and MVC treatment muscle

Supplementary Data 6. Bulk RNA-seq analysis of MuSCs after MVC treatment

Supplementary Data 7. snATAC- and snRNA-seq analysis of TF-gene regulation

Supplementary Data 8. JunB CUT&RUN-seq analysis in mouse MuSCs

Supplementary Data 9. Sequences of oligonucleotides used in the study
